# Antimicrobial Stewardship for Respiratory Pathogens in Swine

**DOI:** 10.3390/antibiotics9110727

**Published:** 2020-10-22

**Authors:** Anna Vilaró, Elena Novell, Vicens Enrique-Tarancón, Jordi Balielles, Eduard Allué, Lorenzo Fraile

**Affiliations:** 1 Grup de Sanejament Porcí, 25192 Lleida, Spain; micro@gsplleida.net (A.V.); elena@gsplleida.net (E.N.); vicens@gsplleida.net (V.E.-T.); jordi@gsplleida.net (J.B.); eduard@gsplleida.net (E.A.); 2 Departament de Ciència Animal, ETSEA, University of Lleida-Agrotecnio, 25198 Lleida, Spain

**Keywords:** antimicrobial susceptibility, swine, respiratory pathogens

## Abstract

The aim of this study was to set up antimicrobial stewardship for swine respiratory pathogens following the recommendation from the European Medicine Agency. The obtained antimicrobial susceptibility pattern recommended using antimicrobial stewardship for each clinical case instead of treatment guidelines focused on pathogens. Thus, the bacteria are isolated and the MIC values, the clinical interpretation for each antimicrobial (susceptible or resistant), additional information about the distance between the MIC obtained and the clinical breakpoint, and set up for each drug, are represented in the report provided for veterinarians. A graph from green (susceptible) to red (resistant) is enclosed for each antimicrobial and microorganism in the report. The greener, the more susceptible is the strain, and the redder, the less susceptible is the strain for each particular antimicrobial. This information could help veterinarians to select the most suitable antimicrobial from first, second, or last option antimicrobials. Thus, veterinarians should choose the antimicrobial, inside each option, with the best antimicrobial susceptibility pattern that corresponds with the greener status in the report. The information provided in the report could be useful for all clinical cases, caused by a certain bacterium within the same pig production system, if an epidemiological link could be established.

## 1. Introduction

*Actinobacillus pleuropneumoniae* (APP), *Pasteurella multocida* (PM), *Mycoplasma hyopneumoniae* (MH), *Bordetella bronchiseptica* (BB), and *Glaesserella (Haemophilus) parasuis* (GP) are the most common bacterial agents involved in the Porcine respiratory disease complex (PRDC) [1,2]. The use of antimicrobials could be necessary to control bacteria involved in PRDC with a therapeutic or metaphylactic (group medication) goal. In particular, the objective of antimicrobial therapy is to provide an effective drug to obtain a quick recovery from the infection in affected animals but reducing the probability of generating antimicrobial resistance [3]. New legislation has been approved for veterinary medicinal products in Europe [4] where special attention has been addressed to antimicrobials. In particular, the selection of the antimicrobial families used in animal production is a key point to meet one-health approach for these drugs. Thus, different antimicrobial families do not have the same risk of generating antimicrobial resistance from a one-health point of view [5]. In this European framework, a recent recommendation from the European Medicine Agency (EMA) [6] comprises the antimicrobials in four categories, from A (avoid), B (restrict), C (caution) to D (prudence) where the selection of one antimicrobial to treat a clinical case must begin with drugs belonging to category D and following with antimicrobials from C and B category, respectively if the treatment fails to cope with the bacterial infection. This recommendation implies that veterinarians must justify the use of antimicrobials and the selection of the antimicrobial to treat one bacterial disease by following prudent use of antimicrobials coming from European directives [7] and associations of practitioners [8]. In veterinary medicine, Antimicrobial Susceptibility Testing (AST) data could predict the clinical outcome of antimicrobial treatment allowing a rational choice of these drugs to treat bacterial infections [3,7]. Antimicrobial susceptibility is usually measured using minimum inhibitory concentration (MIC), which is the lowest antimicrobial concentration that inhibits the growth of the target bacteria in vitro. Moreover, it is necessary to have valid clinical breakpoints (CB) to interpret correctly the MIC value obtained for each clinical case. Thus, all the clinical cases with MIC values below CB could be correctly treated with antimicrobials at the common registered dose. On the other hand, the mutant prevention concentration (MPC) describes a drug concentration threshold or lowest drug concentration blocking the growth of mutant bacterial sub-populations [8,9] that spontaneously arise in bacterial densities of 10^7^–10^9^ colonies, forming units-densities observed with infection. The mutant selection window (MSW) is bordered by the MIC (lower drug concentration) and the MPC (upper drug concentration) [9]. Antibiotic drug concentrations insufficiently inhibiting mutant cell growth result in selective amplification of bacterial cells with reduced drug susceptibility unless dosing to achieve or exceed the MPC and hence prevents the growth of bacterial cells with reduced susceptibility [9,10].

The urgency to use antimicrobials in daily practice seems incompatible with recommendations for prudent use of these drugs [11]. One practical approach could be following the antimicrobial susceptibility pattern described for each clinical case, as well as the prioritization proposed by EMA. In some countries, this antimicrobial stewardship is proposed for each pathogen of veterinary species, but it does not allow justifying the antimicrobial selection for each particular case [12,13,14] as established in the new legislation [4]. In a previous paper of our research group, we have described the antimicrobial susceptibility pattern for some of the most important pig respiratory pathogens in Spain. In this paper, we use this information to carry out antimicrobial stewardship for swine respiratory pathogens, as suggested in the new legislation about antimicrobials in the European Union.

## 2. Results

400 samples were received from wean-to-finish and fattening farms across Spain of sow suffering clinical respiratory cases from 2017 to 2019. Bacterial isolation, as previously described in our former paper [15], was only possible in 80.3% (321/400) of the cases, and in 22% (88/400) of them, it was possible to isolate more than one bacterium. Finally, 162, 130, and 29 strains of APP, PM, and BB were included in this research work because it was not possible to determine the MIC value for GP due to the impossibility for this bacterium to grow with the microdilution technique.

### 2.1. Clusters of Respiratory Pathogens According to Their Antimicrobial Susceptibility

**Figure 1:**
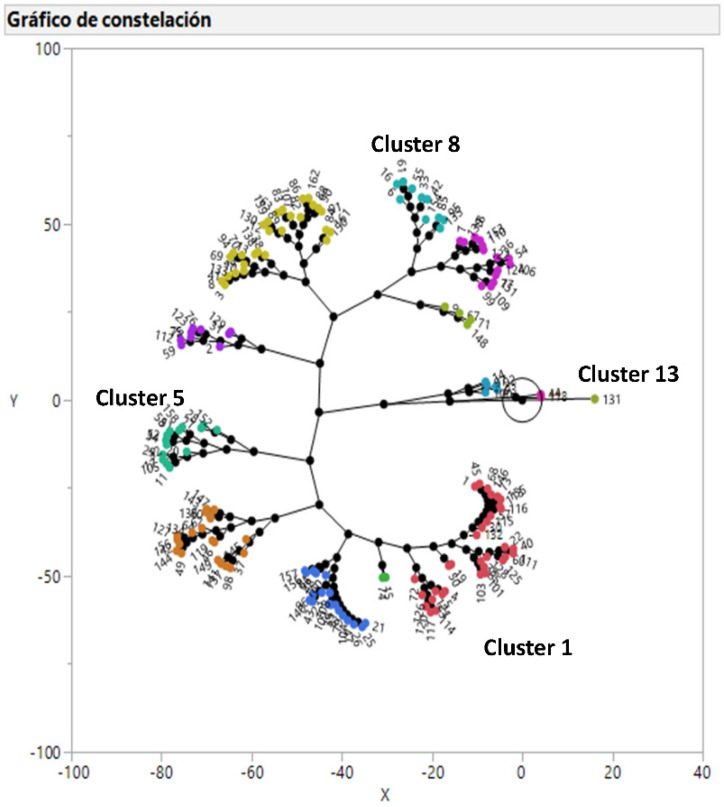
Constellation plot showing the results of 162 *Actinobacillus pleuropneumoniae* isolates after a hierarchical clustering analysis of MIC values for the 10 antimicrobials tested. The numeration begins in cluster 1 and ends in cluster 13 clockwise.

**Figure 2:**
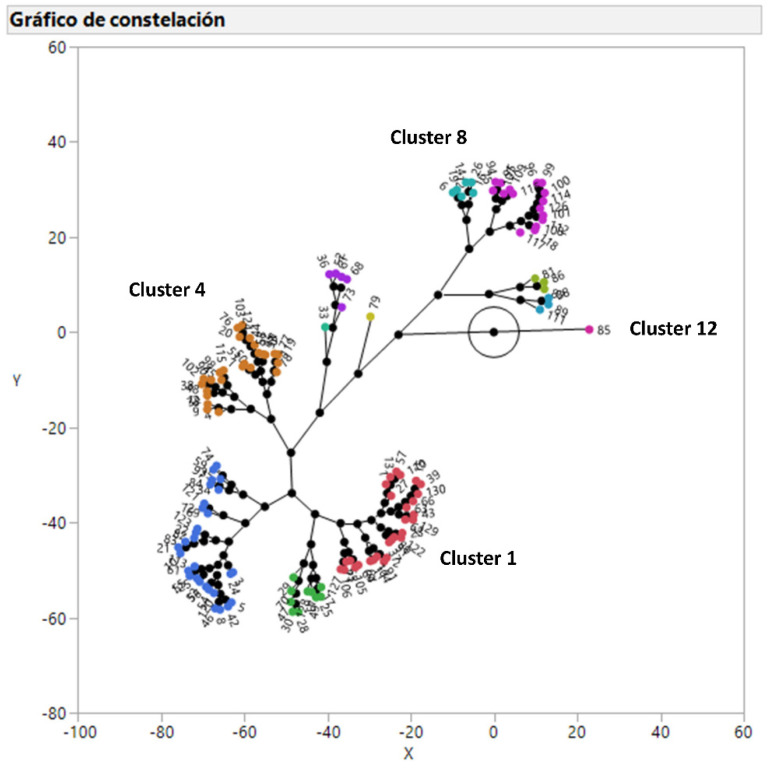
Constellation plot showing the results of 130 *Pasteurella multocida* isolates after a hierarchical clustering analysis of MIC values for the 10 antimicrobials tested. The numeration begins in cluster 1 and ends in cluster 12 clockwise.

*Actinobacillus pleuropneumoniae* and *Pasteurella multocida* isolates were grouped into 13 and 12 clusters, respectively (Figure 1 and Figure 2) and their antimicrobiological susceptibility pattern is detailed is Table 1 and Table 2, respectively. The number of BB strains was too low to carry out a hierarchical clustering analysis. In the case of APP, strains belonging to cluster 1 and 3, including 32.7% of the isolates, can be treated with all the available antimicrobials. Cluster 2, 4, and 11 (16.7% of the isolates) can be treated with all the antimicrobials except with tiamulin, doxycycline, and florfenicol, respectively. On the other hand, cluster 5, 6, 7, 8, and 10 (40.1% of the isolates) cannot be treated with two antimicrobials (Table 1). However, clusters 9, 12, and 13 (10.5% of the isolates) cannot be treated with three antimicrobials. Moreover, cluster 13 (one isolate) shows a peculiar susceptibility pattern with very high MICs for macrolides that is only observed for this isolate. 

In the case of PM, strains belonging to cluster 1 and 10, including 24.6% of the isolates, can be treated with all the available antimicrobials. Cluster 2, 3, and 9 (42.3% of the isolates) can be treated with all the antimicrobials except with doxycycline, tiamulin, and sulfamethoxazole/trimethoprim, respectively. On the other hand, cluster 4, 6, 7, and 8 (29.2% of the isolates) cannot be treated with two families of antimicrobials (Table 2). However, clusters 5, 11, and 12 (3.8% of the isolates) cannot be treated with three families of antimicrobials. Finally, cluster 12 (one isolate) also shows a peculiar susceptibility pattern with very high MICs for macrolides that is unique for this isolate.

### 2.2. Antimicrobial Stewardship for Each Clinical Case

**Figure 3:**
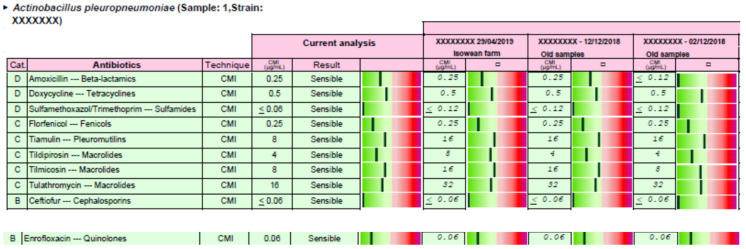
Antimicrobial susceptibility pattern and a pharmacological recommendation based on MIC results, clinical breakpoints available [15], and EMA categorization of antimicrobials [6] are detailed for the case of report 1. It is included the current case and three former APP cases coming from the same sow origin. More information is available in Supplementary Material S1.

**Figure 4:**
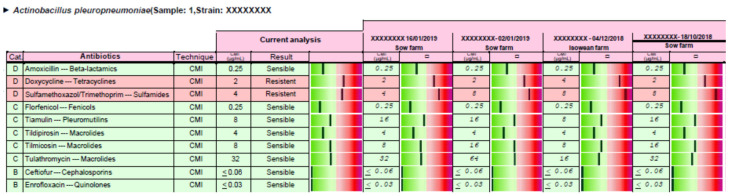
Antimicrobial susceptibility pattern and a pharmacological recommendation based on MIC results, clinical breakpoints available [15], and EMA categorization of antimicrobials [6] are detailed for case of report 2. It is included the current case and three former APP cases coming from the same sow origin. More information is available in Supplementary Material S1.

Antimicrobial stewardship was carried out for each clinical case, and the responsible veterinarian received a report in PDF format by electronic mail. Two examples of the complete reports are available in Supplementary Material S1 (including reports 1 and 2). In the case of report 1, the APP strain could be treated with the 10 antimicrobials tested, but these drugs have been selected according to the EMA categorization of antimicrobials (first, second and last treatment option) and the distance between the MIC value obtained for each antimicrobial versus its clinical breakpoint (Figure 3). Moreover, an order of use has been proposed from first, second, and last option treatment (Table 3). The same procedure has been carried out in the case of report 2, where only 8 out the 10 antimicrobials tested can be used to treat this clinical case (Figure 4 and Table 4).

## 3. Discussion

There is a scarcity of antimicrobial stewardship proposals for swine medicine. The current study aims to address this gap for swine respiratory pathogens taken into account the new legislation about antimicrobials in the European Union and using recently published information of our research group. Thus, Vilaro et al. (2020) has recently established that there are still many options to treat infections by APP and PM with antimicrobials, but the presence of strains resistant to tetracyclines, aminopenicillins, and quinolones in the case of APP and tetracyclines, sulfamides and pleuromutilins in the case of PM should be taken into account to select the most suitable antimicrobial for each clinical case. Moreover, *Bordetella bronchiseptica* was highly susceptible only to tildipirosin and tulathromycin and its susceptibility for florfenicol was close to 50% and <30% for the rest of antimicrobials tested [15].

In the case of APP and PM, 13 and 12 clusters have been defined, respectively, according to their antimicrobial susceptibility pattern using a hierarchical clustering analysis. This multivariate statistical tool allows defining groups with similar antimicrobial susceptibility patterns in a visual and comprehensive way as recently published for *Pasteurella multocida* in swine [16], *Escherichia coli* in humans and animals [17], and uropathogenic *Escherichia coli* in humans [18]. The number of clusters for both bacteria could look very high, taking into account that the antimicrobial susceptibility pattern is favorable for many antimicrobials, but combinations of two or three antimicrobials with low antimicrobial susceptibility generated the observed clusters as published for other microorganisms [16,17,18,19]. The distribution of antimicrobial susceptibility observed for these respiratory pathogens clearly shows that a proper diagnostics and sensitivity testing should be performed for each clinical case. On the other hand, it could be possible to propose general antimicrobial stewardship, not based on a case by case scenario, for any APP and/or PM strain, but this proposal must exclude doxycycline for both bacteria, aminopenicillins, and quinolones for APP and pleuromutilins and sulfamides for PM in order to obtain a good clinical outcome in almost all the clinical cases [15]. Unfortunately, this proposal would discard three antimicrobials (doxycycline, amoxicillin, and sulfamethoxazole/trimethoprim) that should be chosen as first option therapy if the MIC value is below their clinical breakpoints because they belong to D category, according to EMA [6]. For this reason, instead of proposing general antimicrobial stewardship for APP and PM, we propose to carry out a microbiological diagnosis for each clinical case to select the most suitable antimicrobials as first, second and last option therapy according to their MIC value and the European legislation about prudent use of antimicrobials [4]. In the case of BB, the number of antimicrobial options to treat this bacterial infection are very limited [15], and the antimicrobial stewardship based on each clinical case is the only option to select the most suitable antimicrobial treatment. Finally, antimicrobial susceptibility patterns can change with time [20], and it is one of the reasons to monitor it across time. Thus, the proposed antimicrobial stewardship can also tackle this requirement in the long run.

Based on each clinical case, antimicrobial stewardship could be relevant not only to be efficacious from a clinical point of view, but also for the generation of antimicrobial resistance in a dynamic population of microorganisms whose pharmacodynamic properties are continuously evolving. Thus, the selection of an antimicrobial, whose MIC is low and very far from the clinical breakpoint, provides more room to be successful in the treatment of the clinical case even assuming that an incorrect posology regimen (usually underdosing) may take place in drugs administered by oral route in swine medicine [21]. On the other hand, any antimicrobial would reach a concentration in the target tissue, with a higher probability of being above the MPC, if the antimicrobial selected shows a MIC value that is much lower than the clinical breakpoint [22,23]. Taking into account efficacy and antimicrobial resistance issues, we propose to take into account the distance between the MIC observed for each antimicrobial and the clinical breakpoint established for each drug in the antimicrobial stewardship proposal. Thus, veterinarians should choose the antimicrobial, inside each option, with the best antimicrobial susceptibility pattern that corresponds with the greener status in the report described in the result section. This information could help veterinarians to select the most suitable antimicrobial from first, second, or last option antimicrobials in a visual and practical way considering efficacy and antimicrobial resistance criteria.

The antimicrobial stewardship proposed is based on the microbiological isolation and antimicrobial susceptibility determination for each clinical case. This requirement is very difficult to fulfill under field conditions because the microbiological diagnosis and the determination of its antimicrobial susceptibility require between four to six working days. Therefore, the results of the referred clinical case can only be useful for that particular case in the event that the first selected antimicrobial option has not properly worked. Thus, prudent use of antimicrobials has to be carried out, but clinical urgency to select antimicrobials remains. This “irresolvable” problem could have a solution in swine medicine if the concept of epidemiological information is applied for prudent use of antimicrobials. Thus, the microbiological diagnosis and the determination of its antimicrobial sensitivity could be useful for all clinical cases caused by a certain bacterium within the same production system if an epidemiological link can be established. An example could be discussed with a swine actinobacillosis case. Thus, an antimicrobial is selected (according to our experience or from treatment guidelines) to treat animals affected by APP but, at the same time, samples are sent to the laboratory. Thus, the diagnosis will be confirmed, and antimicrobial sensitivity data will be provided between four to six days. From the results obtained, the information will be available to carry out the prudent use of antimicrobials in the following clinical case of swine actinobacillosis coming from the same production system (for example, piglets from the same sow farm). This approach is based on the fact that respiratory pathogens are transmitted from sows to piglets in a vertical way as it has been published for APP [24], PM [25], and other pathogens such as *Staphylococcus aureus* [26,27] and *Streptococcus suis* [28]. This information about antimicrobial susceptibility may be valid for a period of time, depending on local regulations. Obviously, this analysis must be repeated across time to cope with the dynamic population of microorganisms, whose pharmacodynamic properties are continuously evolving. This epidemiological approach has been developed to carry out the prudent use of antimicrobials in pigs for respiratory pathogens at a large scale in Spain since 2018. This initiative has been launched by pig producers and developed by academia beside a diagnostic laboratory specialized in swine medicine (http://www.gsplleida.net/e). Finally, this approach has the acknowledgment of the Spanish Medicine Agency and the Spanish national program to address antimicrobial resistance [29].

## 4. Materials and Methods

### 4.1. Clinical Samples

Samples were drawn from diseased or recently deceased pigs from farms across Spain showing acute clinical signs of respiratory tract infections that had not been exposed to antimicrobial treatment for at least, 15 days before sampling, between the years 2017 and 2019, as detailed in a previous paper of our research group [15].

### 4.2. Bacterial Isolation, Identification, and Antimicrobial Sensitivity Testing

Clinical specimens were cultured aseptically, and identification of isolates (APP, PM, BB, and GP) was carried out by matrix-assisted laser desorption ionization-time of flight (MALDI-TOF Biotyper System, Bruker Daltonics, Bremen, Germany) or VITEK2 (Biomerieux, Marcy l’Etoile, France) as previously described [15]. MIC values were determined using the broth microdilution method by means of customized 96-well microtitre plates (Sensititre, Trek Diagnostic Systems Inc., East Grinstead, UK) containing a total of 10 and 7–8 antimicrobials/concentrations. The antimicrobials tested included amoxicillin, ceftiofur, doxycycline, enrofloxacin, florfenicol, sulfamethoxazole/trimethoprim, tiamulin, tilmicosin, tildipirosin, and tulathromycin [15]. This antimicrobial panel was selected to represent commonly compounds licensed for the treatment of pig respiratory diseases in practice. Briefly, Bacteria were cultured on chocolate agar and incubated at 35–37 °C in ambient air (or with 5–10% CO_2_ for APP) for 18–24 h. Three to five colonies were picked and emulsified in demineralized water (or cation adjusted Mueller–Hinton broth (CAMHB) for APP) to obtain a turbidity of 0.5 McFarland standard (Sensititre™ nephelometer V3011). Suspensions were further diluted in CAMHB (for PM and BB) or veterinary fastidious medium (in the case of APP) to reach a final inoculum concentration of 5 × 10^5^ cfu/mL [15]. Then, the Sensititre panel was reconstituted by adding 100 μL/well of the inoculum. Plates containing PM and BB isolates were incubated at 35 ± 2 °C for 18–20 h. In the case of APP isolates, plates were covered with a perforated seal and incubated at 35 ± 2 °C with 5–10% CO_2_ for 20–24 h. The antibiotic panels were read manually using Sensititre™ Vizion (V2021), and the MIC value was established as the lowest drug concentration inhibiting visible growth. For each strain tested, a colony count and a purity check were performed following CLSI and manufacturer recommendations [15]. Moreover, quality control strains were also included in all susceptibility testing runs. Thus, *Actinobacillus pleuropneumoniae* (ATCC 27090™) and *Escherichia coli* (ATCC 25922™) were included as quality control [15]. The MICs of the quality control strains had to be within acceptable CLSI ranges to accept the results obtained in the laboratory.

### 4.3. Statistical Analysis

A multivariate analysis was applied on the MIC of the 10 antimicrobials for all the strains of APP and PM (enough number of strains). Thus, a constellation plot was generated using between-group linkage via Ward’s hierarchical clustering with JMP^®^, Version 13 (SAS Institute Inc., Cary, NC, USA, 1989–2019) that allows generating clusters of strains according to their antimicrobial susceptibility testing for all the antimicrobials together.

### 4.4. Antimicrobial Stewardship for Each Clinical Case

Antimicrobial stewardship was provided to swine veterinarians for each clinical case, using the clinical breakpoint for each drug and microorganism (susceptible or resistant) previously described in Vilaró et al. (2020) [15] and the categorization of antimicrobials according to European Medicine Agency [6]. Briefly, the bacteria/s isolated, the MIC values, the interpretation for each antimicrobial (susceptible or resistant), and additional information about the distance between the MIC obtained and the clinical breakpoint established for each drug is represented in the report provided. Thus, a graph from green (susceptible) to red (resistant) is enclosed for each antimicrobial and microorganism in the report. The greener, the more susceptible is the strain, and the redder, the less susceptible is the strain for each particular antimicrobial. This information could help veterinarians to select the most suitable antimicrobial from first, second, or last option antimicrobials. Thus, veterinarians should choose the antimicrobial, inside each option, with the best antimicrobial susceptibility pattern that corresponds with the greener status in the report.

## 5. Conclusions

Antimicrobial stewardship is proposed for swine respiratory pathogens in each clinical case based on the determination of antimicrobial susceptibility testing through MIC. This approach allows increasing the probability of success in the treatment and decreasing the probability of generation of antimicrobial resistance. Moreover, an epidemiological approach is proposed to perform a prudent use of antimicrobials instead of using treatment guidelines to better accomplish the new European legislation about veterinary medicinal products. This approach is based on associating the results obtained in an antimicrobial susceptibility determination of a clinical case with an epidemiological link, which could be the sow origin in many cases, to use this information for future clinical cases.

## Figures and Tables

**Table 1:** Antimicrobial susceptibility pattern for the 13 clusters obtained from 162 *Actinobacillus pleuropneumoniae* isolates after a hierarchical clustering analysis. It is detailed in brackets the number of strains belonging to each cluster. Suitable antimicrobials are detailed in green color. The antimicrobials tested included amoxicillin (AMO), ceftiofur (CEF), doxycycline (DOX), enrofloxacin (ENR), florfenicol (FLO), sulfamethoxazole/trimethoprim (SFM), tiamulin (TIA), tilmicosin (TILM), tildipirosin (TILD), and tulathromycin (TUL).

	EMA Category									
	D			C					B	
Cluster	AMO	DOX	SFM	FLO	TIA	TILM	TILD	TUL	CEF	ENR
1 (33)										
2 (2)					NP					
3 (20) **										
4 (20)		NP								
5 (14)		NP	NP							
6 (9)		NP								NP *
7 (28)		NP								NP
8 (10)	NP	NP								
9 (14)	NP	NP								NP
10 (4)	NP	NP *								
11 (5)				NP						
12 (2)		NP	NP							NP
13 (1)	NP	NP				NP	NP	NP		

* Extremely high MIC values. ** MIC values for amoxicillin are lower than for cluster 1. NP (not possible and highlighted in red) means that the MIC value excludes this antimicrobial for the treatment of the bacterial infection at the registered dose. A recent recommendation from the European Medicine Agency (EMA) [6] comprises the antimicrobials in four categories, from A (avoid), B (restrict), C (caution) to D (prudence).

**Table 2:** Antimicrobial susceptibility pattern for the 12 clusters obtained from 130 *Pasteurella multocida* isolates after a hierarchical clustering analysis. It is detailed in brackets the number of strains belonging to each cluster. Suitable antimicrobials are detailed in green color. The antimicrobials tested included amoxicillin (AMO), ceftiofur (CEF), doxycycline (DOX), enrofloxacin (ENR), florfenicol (FLO), sulfamethoxazole/trimethoprim (SFM), tiamulin (TIA), tilmicosin (TILM), tildipirosin (TILD), and tulathromycin (TUL).

	EMA Category									
	D			C					B	
Cluster	AMO	DOX	SFM	FLO	TIA	TILM	TILD	TUL	CEF	ENR
1 (29)										
2 (10)		NP								
3 (29)					NP					
4 (26)		NP			NP					
5 (1)	NP	NP			NP					
6 (5)		NP			NP *					
7 (1)	NP *	NP								
8 (6)			NP		NP					
9 (16)			NP							
10 (3)										**
11 (3)		NP			NP					NP
12 (1)		NP				NP	NP	NP		NP

* Extremely high MIC values. ** Higher MIC values than for cluster 1. NP (not possible and highlighted in red) means that the MIC value excludes this antimicrobial for the treatment of the bacterial infection at the registered dose. A recent recommendation from the European Medicine Agency (EMA) [6] comprises the antimicrobials in four categories, from A (avoid), B (restrict), C (caution), to D (prudence).

**Table 3:** Antimicrobial stewardship for an *Actinobacillus pleuropneumoniae* strain isolated in a clinical case with reference report 1.

Antimicrobial Stewardship				
Clinical Case	Antimicrobial	EMA Category	Order of Use Proposed	Suggestion Inside Each Option
*Actinobacillus pleuropneumoniae* UP2071504				
	Sulfamethoxazole/trimethropim	D	First option	1
	Amoxicillin	D	First option	2
	Doxycycline	D	First option	3
	Florfenicol	C	Second option	1
	Tildipirosin/tulathromycin	C	Second option	2
	Tiamulin/tilmicosin	C	Second option	3
	Ceftiofur	B	Last option	1
	Enrofloxacin	B	Last option	2

A recent recommendation from the European Medicine Agency (EMA) [6] comprises the antimicrobials in four categories, from A (avoid), B (restrict), C (caution), to D (prudence).

**Table 4:** Antimicrobial stewardship for an *Actinobacillus pleuropneumoniae* strain isolated in a clinical case with reference report 2.

Antimicrobial Stewardship				
Clinical Case	Antimicrobial	EMA Category	Order of Use Proposed	Suggestion Inside Each Option
*Actinobacillus pleuropneumoniae* UG1970383				
	Amoxicillin	D	First option	1
	Florfenicol	C	Second option	1
	Tildipirosin	C	Second option	2
	Tiamulin/tilmicosin/tulathromycin	C	Second option	3
	Ceftiofur/enrofloxacin	B	Last option	1

A recent recommendation from the European Medicine Agency (EMA) [6] comprises the antimicrobials in four categories, from A (avoid), B (restrict), C (caution), to D (prudence).

## Supplementary Materials

The following are available online at https://www.mdpi.com/2079-6382/9/11/727/s1, S1: Report for the veterinarian of clinical cases 1 and 2. This report summarizes the microbiological results, the antimicrobial susceptibility pattern, and a pharmacological recommendation based on MIC results, clinical breakpoints available [15], and EMA categorization of antimicrobials [6]. It includes the current case and three and four former APP cases coming from the same sow origin for case 1 and 2, respectively.
