# Supplementary material for: Antimicrobial Stewardship for Respiratory Pathogens in Swine
Source: Antibiotics (Basel). 2020 Oct 22;9(11):727. doi: 10.3390/antibiotics9110727 (PMC7690587; doi:10.3390/antibiotics9110727)
Supplement: Supplementary file 1 [file antibiotics-09-00727-s001.pdf]

Associació Porcsa
Grup de Sanejament
Porcí de Lleida
GSP
Partida La Caparrella, 97-C
25192 LLEIDA
www.gsplleida.net
Tel. 973 26 92 29 / Fax 973 27 82 10

Results report

Farm data:

**Farm Code:**	**Secondary id**	**REGA Id:**	
**Owner**		**Species**	Swine
**Village:**		**Region:**	
**Veterinarian:**		**Farm name:**	

Registry data:

**Registry Id:**	**Report 1**	**Num. of samples**	1
**Registry date**	16/04/2020	**Date of samples:**	15/04/2020
**D. Public.:**	22/04/2020	**D. Public. Partial:**	

Observations

Microbiology

Epidemiological link:

Sows farm: **Transition farm:**

Microbiology

· Isolation and identification

Strain	Sample	Sample description	Type	rial identification m	Result	Microorganism
XXXXXXXX	1	1	Lung	MALDI-TOF	Growth	*Actinobacillus pleuropneumoniae*

Serotype APP

Strain	APP1	APP2	APP3	APP4	APP5	APP6	APP7	APP8	APP9/11	APP10	APP12	APP13	APP14	APP15	APP16	APP17	APP18
XXXXXXXX	NEG	NEG	NEG	NEG	NEG	NEG	NEG	NEG	POS	NEG	NEG	NEG	NEG	NEG	NEG	NEG	NEG

Antimicrobial susceptibility

► ***Actinobacillus pleuropneumoniae*** **(Sample: 1,Strain: XXXXXXX)**

			Current analysis			XXXXXXXX 29/04/2019 Isowean farm		XXXXXXXX - 12/12/2018 Old samples		XXXXXXXX - 02/12/2018 Old samples			
Cat.	Antibiotics	Technique	CMI (µg/mL)	Result		CMI (µg/mL)	□	CMI (µg/mL)	□	CMI (µg/mL)	□	CMI (µg/mL)	
D	Amoxicillin --- Beta-lactamics	CMI	0.25	Sensible		0.25		0.25		≤ 0.12		–	
D	Doxycycline --- Tetracyclines	CMI	0.5	Sensible		0.5		0.5		0.5		–	
D	Sulfamethoxazol/Trimethoprim --- Sulfamides	CMI	≤ 0.06	Sensible		≤ 0.12		≤ 0.12		≤ 0.12		–	
C	Florfenicol --- Fenicols	CMI	0.25	Sensible		0.25		0.25		0.25		–	
C	Tiamulin --- Pleuromutilins	CMI	8	Sensible		16		16		16		–	
C	Tildipirosin --- Macrolides	CMI	4	Sensible		8		4		4		–	
C	Tilmicosin --- Macrolides	CMI	8	Sensible		16		16		8		–	
C	Tulathromycin --- Macrolides	CMI	16	Sensible		32		32		32		–	
B	Ceftiofur --- Cephalosporins	CMI	≤ 0.06	Sensible		≤ 0.06		≤ 0.06		≤ 0.06		–	

Associació Porcsa. Grup de Sanejament Porcí de Lleida. Partida La Caparrella, 97C - 25192 Lleida - Nif. G25340589

Associació Porcsa<br>Grup de Sanejament<br>Porcí de Lleida<br>GSP	Partida La Caparrella, 97-C<br>25192 LLEIDA<br>www.gsplleida.net<br>Tel. 973 26 92 29 / Fax 973 27 82 10

Results report

Farm data:

**Farm Code:**	**Secondary id**	**REGA Id:**	
**Owner**		**Species**	Swine
**Village:**		**Region:**	
**Veterinarian:**		**Farm name:**	

Registry data:

**Registry Id:**	**Report 1**	**Num. of samples**	1
**Registry date**	16/04/2020	**Date of samples:**	15/04/2020
**D. Public.:**	22/04/2020	**D. Public. Partial:**	

► ***Actinobacillus pleuropneumoniae*(Sample: 1,Strain: XXXXXXXX)**

			**Current analysis**			**XXXXXXXX - 29/04/2019 Isowean farm**		**XXXXXXXX - 12/12/2018 Old samples from curre**		**XXXXXXXX- 02/12/2018 Old samples from curre**		-	
Cat.	**Antibiotics**	Technique	CMI (µg/mL)	Result		CMI (µg/mL)	□	CMI (µg/mL)	□	CMI (µg/ml)	□	CMI (µg/ml)	-
B	Enrofloxacin --- Quinolones	CMI	0.06	Sensible		0.06		0.06		0.06		-	-

The greener, the more susceptible is the strain and the redder, the less susceptible is the strain for each particular antimicrobial. The MIC of each drug and microorganism is interpreted as susceptible or resistant depending if this particular strain can be treated or not, respectively with a veterinary medicinal product containing the antimicrobial at the registered dose. A clinical breakpoint is used to do so. Additional information about the distance between the MIC obtained and the clinical breakpoint established for each drug is represented. Thus, a graph from green (susceptible) to red (resistant) is enclosed for each antimicrobial in the report.

There is also a recent recommendation of EMA and AEMPS that now comprises four categories of antimicrobials, from A (avoid), B (restrict), C (caution) to D (prudence). If it is taken into account the legislation about prudent use of antimicrobials, Category D antimicrobials should be used as first therapeutic option. If these drugs are unable to solve the clinical case. category C antimicrobials should be used as second option. If all the them are unable to solve the clinical case, Category B antimicrobials can be chosen as last therapeutic option.

Pharmacological interpretation

The strain of *Actinobacillus pleuropneumoniae* is susceptible to eight family of antimicrobials. Thus, the treatment is probable to be eficacious with aminopenicillins (amoxicillin), tetracyclines (doxycyline), cefalosporins (ceftiofur), macrolides (tilmicosin, tulathromycin and tildipirosin), pleuromutilins (tiamulin), quinolones (enrofloxacin), fenicols (florfenicol) and sulfamides. (sulfamethoxazole/trimethropim). If it is taken into account the legislation about prudent use of antimicrobials, sulfamides , tetracyclines and aminopenicicillins should be used as first therapeutic option (Category D). If these families are unable to solve the clinical case, fenicoles, macrolides and pleuromutilins should be used as second therapeutic option. If all the them are unable to solve the clinical case, cefalosporins and quinolones can be chosen as last therapeutic option (category B).

Anna Vilaró Vives
Responsable técnica. Laboratorio Microbiología
E-mail: micro@gsplleida.net

Lorenzo José Fraile Sauce
Profesor Agregado UdL
E-mail: Lorenzo.fraile@ca.udl.cat

Laboratory recognized by the Generalitat de Catalunya. Departament d'Agricultura, Ramaderia, Pesca i Alimentació. Direcció General de Producció, Innovació i Indústries Agroalimentàries.
The results in this analysis report are only about the samples under control.
If the GSP don't allow, this report can't be reproduced, if will not completely.

Associació Porcsa. Grup de Sanejament Porcí de Lleida. Partida La Caparrella, 97C - 25192 Lleida - Nif. G25340589

Associació Porcsa
Grup de Sanejament
Porcí de Lleida
GSP
Partida La Caparrella, 97-C
25192 LLEIDA
www.gsplleida.net
Tel. 973 26 92 29 / Fax 973 27 82 10

Results report

Farm data:

**Farm Code:**	**Secondary id**	**REGA Id:**	
**Owner**		**Species**	Swine
**Village:**		**Region:**	-
**Veterinarian:**		**Farm name:**	

Registry data:

**Registry Id:**	**Report 2**	**Num. of samples**	1
**Registry date**	22/02/2019	**Date of samples:**	21/02/2019
**D. Public.:**	07/03/2019	**D. Public. Partial:**	

Observations

Microbiology

Epidemiological link:

Sows farm: Transition farm:

Microbiology

· Isolation and identification

Strain	Sample	Sample description	Type	rial identification m	Result	Microorganism
XXXXXXXX	1	1	Lung	Vitek	Growth	*Actinobacillus pleuropneumoniae*

Antimicrobial susceptibility

► ***Actinobacillus pleuropneumoniae*****(Sample: 1,Strain: XXXXXXXX**

Cat.	Antibiotics	Technique	Current analysis CMI (µg/mL)	Current analysis Result		XXXXXXXX 16/01/2019 Sow farm CMI (µg/mL)	□	XXXXXXXX- 02/01/2019 Sow farm CMI (µg/mL)	□	XXXXXXXX - 04/12/2018 Isowean farm CMI (µg/mL)	□	XXXXXXXX- 18/10/2018 Sow farm CMI (µg/mL)	□
D	Amoxicillin --- Beta-lactamics	CMI	0.25	Sensible		0.25		0.25		0.25		0.25	
D	Doxycycline --- Tetracyclines	CMI	2	Resistent		2		2		4		2	
D	Sulfamethoxazol/Trimethoprim --- Sulfamides	CMI	4	Resistent		4		8		8		8	
C	Florfenicol --- Fenicols	CMI	0.25	Sensible		0.25		0.25		0.25		0.25	
C	Tiamulin --- Pleuromutilins	CMI	8	Sensible		16		16		8		16	
C	Tildipirosin --- Macrolides	CMI	4	Sensible		4		4		4		4	
C	Tilmicosin --- Macrolides	CMI	8	Sensible		8		16		8		16	
C	Tulathromycin --- Macrolides	CMI	32	Sensible		32		64		16		32	
B	Ceftiofur --- Cephalosporins	CMI	$\leq$ 0.06	Sensible		$\leq$ 0.06		$\leq$ 0.06		$\leq$ 0.06		$\leq$ 0.06	
B	Enrofloxacin --- Quinolones	CMI	$\leq$ 0.03	Sensible		$\leq$ 0.03		$\leq$ 0.03		$\leq$ 0.03		$\leq$ 0.03	

Associació Porcsa. Grup de Sanejament Porcí de Lleida. Partida La Caparrella, 97C - 25192 Lleida - Nif. G25340589

Associació Porcsa
Grup de Sanejament
Porcí de Lleida
GSP
Partida La Caparrella, 97-C
25192 LLEIDA
www.gsplleida.net
Tel. 973 26 92 29 / Fax 973 27 82 10

Results report

Farm data:

**Farm Code:**	**Secondary id**	**REGA Id:**	
**Owner**		**Species**	Swine
**Village:**		**Region:**	-
**Veterinarian:**		**Farm name:**	

Registry data:

**Registry Id:**	**Report 2**	**Num. of samples**	1
**Registry date**	22/02/2019	**Date of samples:**	21/02/2019
**D. Public.:**	07/03/2019	**D. Public. Partial:**	

The greener, the more susceptible is the strain and the redder, the less susceptible is the strain for each particular antimicrobial. The MIC of each drug and microorganism is interpreted as susceptible or resistant depending if this particular strain can be treated or not, respectively with a veterinary medicinal product containing the antimicrobial at the registered dose. A clinical breakpoint is used to do so. Additional information about the distance between the MIC obtained and the clinical breakpoint established for each drug is represented. Thus, a graph from green (susceptible) to red (resistant) is enclosed for each antimicrobial in the report.

There is also a recent recommendation of EMA and AEMPS that now comprises four categories of antimicrobials, from A (avoid), B (restrict), C (caution) to D (prudence). If it is taken into account the legislation about prudent use of antimicrobials, Category D antimicrobials should be used as first therapeutic option. If these drugs are unable to solve the clinical case. category C antimicrobials should be used as second option. If all the them are unable to solve the clinical case, Category B antimicrobials can be chosen as last therapeutic option.

Associació Porcsa. Grup de Sanejament Porcí de Lleida. Partida La Caparrella, 97C - 25192 Lleida - Nif. G25340589

Pharmacological interpretation

The strain of *Actinobacillus pleuropneumoniae* is susceptible to six family of antimicrobials. Thus, the treatment is probable to be eficacious with aminopenicillins (amoxicillin), cefalosporins (ceftiofur), macrolides (tilmicosin, tulathromycin and tildipirosin), pleuromutilins (tiamulin), quinolones (enrofloxacin and marbofloxacin), and fenicols (florfenicol). If it is taken into account the legislation about prudent use of antimicrobials, aminopenicicillins should be used as first therapeutic option (Category D). If this family is unable to solve the clinical case, fenicoles, macrolides and pleuromutilins should be used as second therapeutic option. If all the them are unable to solve the clinical case, cefalosporins and quinolones can be chosen as last therapeutic option (category B).

0€

Anna Vilaró Vives
Responsable técnica. Laboratorio Microbiología
E-mail: micro@gsplleida.net

Lorenzo José Fraile Sauce
Profesor Agregado UdL
E-mail: Lorenzo.fraile@ca.udl.cat
